# Effects of Bilateral Assistance for Hemiparetic Gait Post-Stroke Using a Powered Hip Exoskeleton

**DOI:** 10.1007/s10439-022-03041-9

**Published:** 2022-08-13

**Authors:** Yi-Tsen Pan, Inseung Kang, James Joh, Patrick Kim, Kinsey R. Herrin, Trisha M. Kesar, Gregory S. Sawicki, Aaron J. Young

**Affiliations:** 1 Woodruff School of Mechanical Engineering, Georgia Institute of Technology, Atlanta, GA 30332 USA; 2 Department of Brain and Cognitive Sciences, Massachusetts Institute of Technology, Cambridge, MA 02139 USA; 3 School of Electrical and Computer Engineering, Georgia Institute of Technology, Atlanta, GA 30332 USA; 4 Institute for Robotics and Intelligent Machines, Georgia Institute of Technology, Atlanta, GA 30332 USA; 5 Division of Physical Therapy, Emory University, Atlanta, GA 30322 USA; 6 Department of Rehabilitation Medicine, Emory University, Atlanta, GA 30307 USA; 7 School of Biological Science, Georgia Institute of Technology, Atlanta, GA 30332 USA

**Keywords:** Powered exoskeleton, Stroke gait, Hip assistance, Walking speed, Biomechanics

## Abstract

Hemiparetic gait due to stroke is characterized by an asymmetric gait due to weakness in the paretic lower limb. These inter-limb asymmetries increase the biomechanical demand and reduce walking speed, leading to reduced community mobility and quality of life. With recent progress in the field of wearable technologies, powered exoskeletons have shown great promise as a potential solution for improving gait post-stroke. While previous studies have adopted different exoskeleton control methodologies for restoring gait post-stroke, the results are highly variable due to limited understanding of the biomechanical effect of exoskeletons on hemiparetic gait. In this study, we investigated the effect of different hip exoskeleton assistance strategies on gait function and gait biomechanics of individuals post-stroke. We found that, compared to walking without a device, powered assistance from hip exoskeletons improved stroke participants’ self-selected overground walking speed by 17.6 ± 2.5% and 11.1 ± 2.7% with a bilateral and unilateral assistance strategy, respectively (*p* < 0.05). Furthermore, both bilateral and unilateral assistance strategies significantly increased the paretic and non-paretic step length (*p* < 0.05). Our findings suggest that powered assistance from hip exoskeletons is an effective means to increase walking speed post-stroke and tuning the balance of assistance between non-paretic and paretic limbs (i.e., a bilateral strategy) may be most effective to maximize performance gains.

**Supplementary Information:**

The online version contains supplementary material available at 10.1007/s10439-022-03041-9.

## Introduction

Powered exoskeletons hold great promise in augmenting humans for improved mobility.^35, 44^ These exoskeletons assist in specific phases of the gait cycle to enhance the physical capabilities of people with lower limb disabilities.^22, 32, 47^ One of the leading clinical populations that can potentially benefit from these powered robotic exoskeletons is individuals with hemiparetic gait following a stroke. Stroke survivors often exhibit muscle weakness and impaired coordination in the paretic ankle.^42^ This causes a compensatory action by the proximal hip joint leading to an abnormal and asymmetric gait pattern.^5, 8^ Due in part^27^ to pronounced gait asymmetry, stroke survivors often experience an increase in metabolic and biomechanical demands, limiting community mobility and leading to a reduced quality of life.^21^

Because stroke survivors exhibit weakness in ankle plantar flexor muscles, research groups have previously utilized ankle exoskeletons to improve their walking speed.^1, 24, 40^ While some studies have shown positive results, effects of ankle exoskeletons on stroke gait were highly varied, in part due to users not being able to fully utilize the exoskeleton's assistance to increase their walking speed. During gait, ankle push-off is a critical phase that facilitates a smooth stance to swing transition and directly relates to the forward propulsion of the body's center of mass. Specifically, the anterior ground reaction force (AGRF), a measure of push-off, is correlated to walking speed^9^ and gait asymmetry.^2^ However, hemiparetic gait due to post-stroke exhibits suboptimal limb posture and orientation (e.g., reduced hip extension angle) during the propulsive push-off phase, making it challenging to optimize ankle assistance timing. Indeed, recent studies showed that even with advanced controllers, ankle exoskeleton joint torque is often translated into vertical instead of anteriorly-directed ground reaction forces.^24^ This may be the fundamental limitation of using an ankle exoskeleton, where the source of powered assistance is located at the distal joint. Instead, a potential solution is to utilize a powered hip exoskeleton and provide assistance at the proximal joint. This approach allows the assistive joint torque to drive hip extension during the swing to stance transition (0–20% of the gait cycle) when the user’s trailing limb is in a position with better mechanical leverage, producing a greater AGRF during the push-off phase.

Several exoskeleton studies have shown exciting results in augmenting humans by assisting at the hip joint.^7, 17, 20, 36, 46, 48^ However, most of these studies were conducted on able-bodied adults, which may not directly translate to stroke gait. Buesing *et al*.^4^ evaluated the effect of using a powered hip exoskeleton on stroke gait and showed that after 6–8 weeks of functional gait training using the device, the participants improved self-selected walking speed and spatiotemporal gait parameters such as paretic side swing time and stride length. Similarly, Lee *et al*.^18^ showed that while using a powered hip exoskeleton device, participants with stroke not only improved the baseline walking speed and spatiotemporal gait parameters but also reduced the metabolic cost of walking. Yoshimoto *et al*.^43^ demonstrated significantly greater improvements in walking speed compared to the control group following an 8-week training protocol with a powered hip-knee exoskeleton.

While these few studies showed a potential to use hip exoskeletons for improving stroke gait, there is limited information linking exoskeleton assistance strategies or parameters to biomechanical outcomes during walking. For example, most studies that examined the effects of hip exoskeletons on stroke gait employed a unilateral assistance strategy (which may not capture the exoskeleton's full capability). Furthermore, these studies did not carefully explore the underlying changes in the stroke survivor’s biomechanical mechanisms when walking with an exoskeleton system.^3, 38, 43^ Finally, exoskeleton studies that used a bilateral hip assistance strategy on stroke gait were also limited because the applied control framework was based on the symmetric walking pattern of able-bodied users.^4, 12, 15, 18, 25^ Thus, there is a fundamental need for a better understanding of how different powered hip exoskeleton assistance strategies impact the gait biomechanics of individuals post-stroke.

In this study, we investigated the biomechanical effects of bilateral powered hip exoskeleton assistance on hemiparetic gait due to stroke. Our central hypothesis was that stroke survivors using a powered hip exoskeleton that applies bilateral assistance (paretic + non-paretic limbs) would increase self-selected walking speed more than unilateral assistance strategies (paretic or non-paretic limb only). Our underlying rationale was that additional hip assistance on the non-paretic side can further increase the user's step length, leading to an increased AGRF for a greater propulsive impulse which has a direct correlation with walking speed. To test this hypothesis, we measured the stroke survivor’s overground self-selected walking speed while wearing a powered hip exoskeleton with different assistance strategies across a range of assistance magnitudes. Our study findings will direct and move the field forward in understanding optimized control approaches, allowing to design personalized hip exoskeleton control systems that can increase mobility, leading to an improved quality of life for stroke survivors.

## Materials and Methods

### Exoskeleton Hardware

We utilized a lightweight, autonomous, powered hip exoskeleton that can provide bilateral hip flexion and extension assistance in the sagittal plane, Gait Enhancing and Motivating System (Samsung Electronics, South Korea). The device has an additional passive joint at the hip for free abduction and adduction in the frontal plane. The device has a suite of on-board mechanical sensors including encoders at each hip joint and an inertial measurement unit located at the pelvis, that enable real-time measurements of the user's kinematics (e.g., hip joint position) during locomotion. To ensure better wearability and suspension of the device, we attached an additional thoracolumbar interface to the hardware (total exoskeleton weight of 3.3 kg).

### Biological Torque Controller

**Figure 1:**
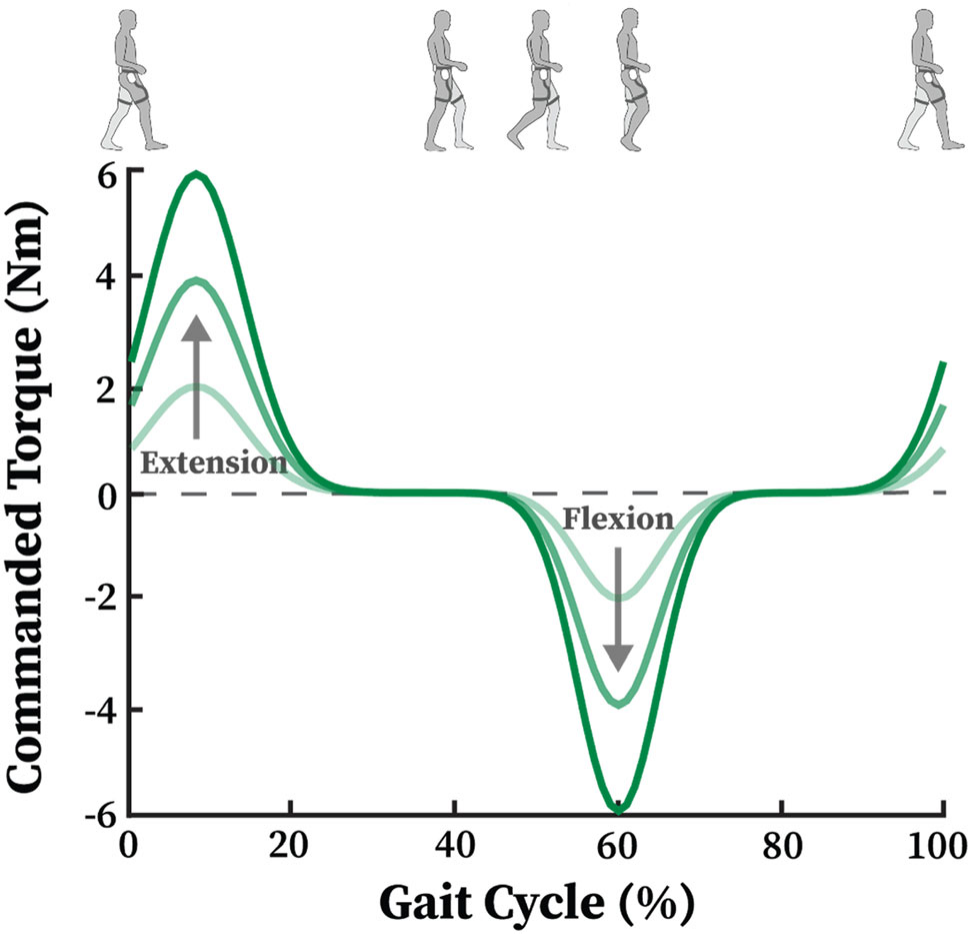
Biological torque controller used for providing bilateral hip assistance. A sum of univariate Gaussian curves was used to generate the desired assistance profile. Assistance level can be tuned to provide desired peak joint torque magnitude during the gait cycle.

We utilized a biological torque controller previously presented^14^ (Fig. 1) to ensure consistent and continuous assistance throughout the gait cycle. The onset and peak assistance timing of hip flexion and extension were based on the values from our previous hip exoskeleton study, which showed the largest metabolic cost reduction for able-bodied subjects.^45^ The onset timing for hip flexion and extension assistance was set to 45 and 90% of the gait cycle, respectively (defining heel strike as 0%). The onset timings tuned for each subject were bounded within ± 5% to the initial starting values. The peak timing for hip flexion and extension was set to 60% and 10% of the gait cycle, respectively. To generate a desired assistance profile, we used a sum of univariate Gaussian curves where the mean and the variance were related to the assistance timing parameters. To estimate the user's gait phase, we utilized the hip joint position as an event marker to detect the maximum hip flexion event which corresponds to 90% of the gait cycle.^7^ We calculated the time since the most recent hip extension event divided by the average stride duration computed from previous two gait cycles.

### Participants and Inclusion/Exclusion Criteria

Five participants with chronic stroke (≥ 6 months post-stroke) and subsequent unilateral hemiparesis were recruited (2 females; age: 52.4 ± 10.2 years; time post-stroke: 64 ± 28 months; heights: 172.5 ± 9.8 cm; weights: 74.5 ± 13 kg) for this study (Table 1). The inclusion criteria for this study were: (1) at least 6 months post-stroke, (2) age 18–85 years, (3) able to walk without support with a preferred walking speed ≥ 0.4 m/s for at least 6 min, and (4) have adequate cognitive level with a Mini-Mental State Examination score ≥ 17.

**Table 1:** Participant demographics.

Subject	Age	Gender	Height (cm)	Weight (kg)	Paretic side	Assistance range (Nm/kg)	Time post stroke	Assistive device	Free speed (m/s)	Fastest SSWS (m/s) from the Exo-assisted condition	
ST01	37	M	180.5	89.0	Right	0.02–0.07	6 yrs, 3 mo	AFO	0.60^a^	0.81	N3P2
ST02	57	M	175.5	81.0	Left	0.02–0.07	9 yrs, 5 mo	FES	0.86^b^	1.04	N2P3
ST03	55	M	183.0	84.8	Right	0.02–0.07	3 yrs, 1 mo	FES^*^	0.58^a^	0.77	N3P0
ST04	67	F	156.0	57.9	Left	0.04–0.10	3 yrs, 2 mo		0.94^b^	1.18	N3P1
ST05	46	F	167.5	59.9	Right	0.03–0.10	4 yrs, 7 mo		1.01^b^	1.15	N3P0

Functional walking category based on self-selected walking speed:^34^ household ambulators: < 0.4 m/s

^a^Limited community ambulators: 0.4–0.8 m/s

^b^Community ambulators: > 0.8 m/s

^*^Knee brace on the non-paretic side

The exclusion criteria of this study were: (1) any medical issues that can significantly influence gait such as cardiopulmonary or respiratory disorders, (2) severe spasticity of the lower limb muscles (Modified Ashworth Scale > 3), and (3) pre-existing neurological disorders other than stroke (e.g., Parkinson's disease or dementia). An additional screening process regarding inclusion and exclusion criteria was performed by the trained clinician onsite at our research facility. The study was approved by the Georgia Institute of Technology Institutional Review Board and informed written consent was obtained for all subjects.

### Experimental Protocol

**Figure 2:**
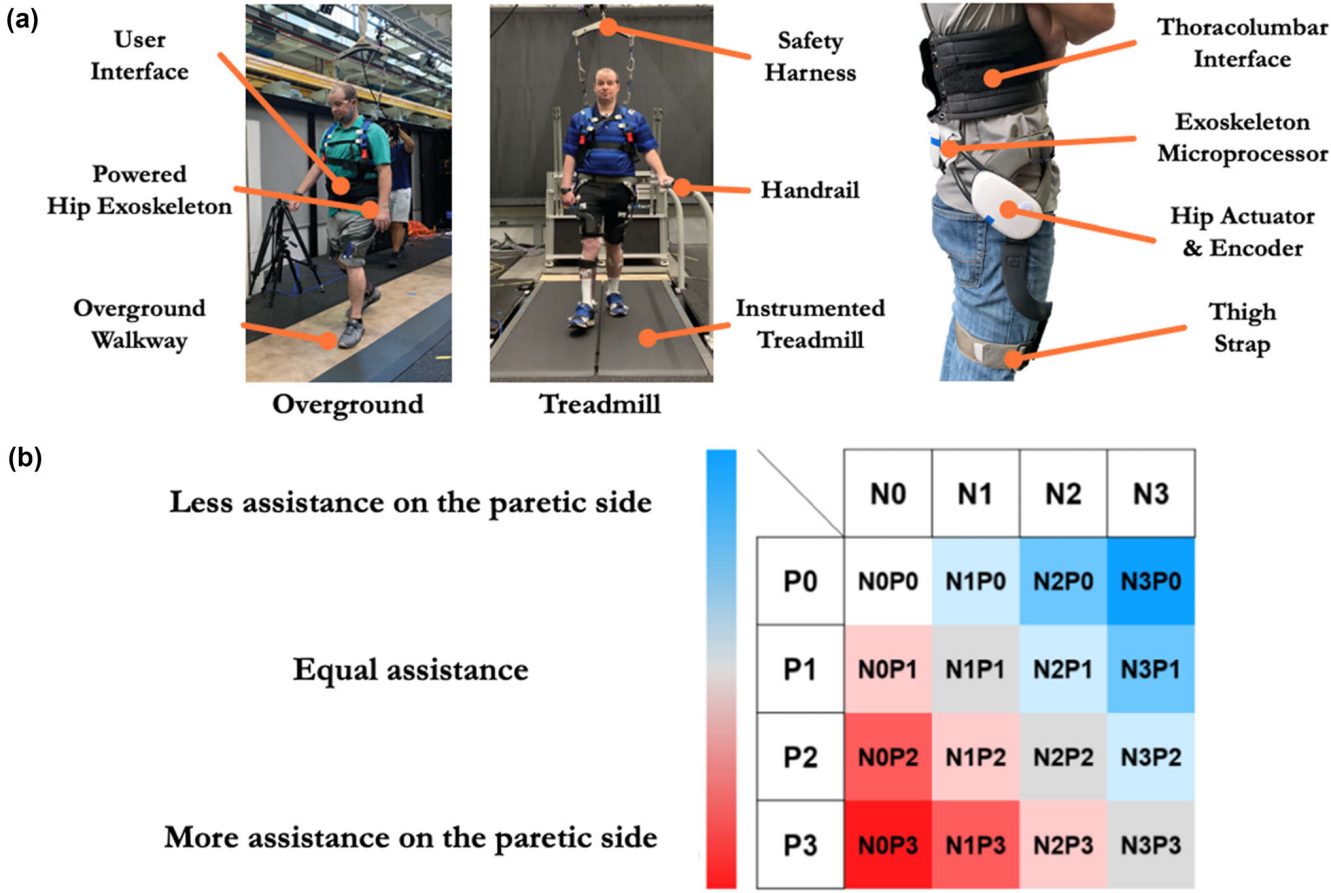
Experimental setup for testing human participants. (a) (left) Participants walked on a treadmill and overground walkway while wearing a powered hip exoskeleton. (right) The powered hip exoskeleton consists of actuators and on-board sensors that allow hip flexion/extension assistance during walking. (b) Experimental conditions applied many different exoskeleton assistance strategies. P and N corresponds to paretic and non-paretic side and assistance level of 0, 1, 2, and 3 refers to 0 Nm, 2 Nm, 4 Nm, and 6 Nm, respectively. Colors indicate the symmetry of exoskeleton assistance. Red is paretic side focused. Blue is non-paretic side focused.

The experimental protocol consisted of three sessions: (1) baseline assessment, (2) exoskeleton fitting and controller parameter tuning, and (3) exoskeleton walking trials. During Session 1, the participant’s gait characteristics without wearing the exoskeleton were measured. Comfortable overground walking speed and spatiotemporal gait parameters were measured using a 6-m walkway (Zeno, ProtoKinetics, Havertown, PA). Subjects were asked to complete × 4 passes across the walkway at their self-selected walking speed. Later, subjects were asked to walk on an instrumented split-belt treadmill (Bertec Corporation, Columbus, Ohio). The treadmill speed was set at 80% of the overground self-selected walking speed (Table 1). Subjects walked on the treadmill for 1 min while the ground reaction force data were collected. During Session 2, the subjects donned and were fitted to the exoskeleton with an adjustable waist belt, thigh interface, and thigh straps (Fig. 2a). During this procedure, subjects first acclimated to the exoskeleton by walking on the treadmill with the device zero torque mode to ensure there was no discomfort. Following the initial familiarization phase, we provided hip assistance bilaterally by setting the assistance magnitude to a medium level (4 Nm). We utilized this magnitude to tune the assistance timing parameters for each side while the subject was walking on the treadmill. During this tuning phase, we fixed the hip flexion and extension assistance duration to a nominal value that was shown to be effective in our previous study.^13^ The hip flexion and extension onset timings were tuned based on the assessment by a clinician and the subject's verbal feedback to ensure that the exoskeleton assistance was congruent with the subject's limb movement and did not induce further gait deviations. The hip flexion and extension onset timings for each side were set the same for all assistance magnitude conditions (2 Nm, 4 Nm, and 6 Nm) in Session 3.

In Session 3, the participants were asked to walk with the exoskeleton with 16 different combinations of assistance magnitude for both the paretic and non-paretic sides. The assistance condition was noted as level 0 (no assistance), 1 (low), 2 (medium), and 3 (high) with the corresponding exoskeleton magnitude set to 0 Nm, 2 Nm, 4 Nm, and 6 Nm, respectively (Fig. 2b). For example, in the N0P1 condition, the exoskeleton assistance was only provided to the paretic side with a magnitude set to 2 Nm. For each assistance condition, the subject walked on the treadmill for 1 min. Following the treadmill trial, the subjects completed 4 passes on the 6-m walkway with the same assistance condition. Throughout Session 3, the order of these assistance conditions was randomized. To ensure safety, all subjects wore an overhead safety harness during the entire experiment and a handrail was mounted on the subject's non-paretic side during treadmill walking.

### Data Collection and Analysis

To evaluate gait functions under different exoskeleton assistance conditions post-stroke, we recorded the subject's walking speed, step length, and ground reaction force from the instrumented treadmill. The subjects’ overground walking speed, step length, and step length asymmetry were measured and processed using the Movement Analysis software (ProtoKinetics, Havertown,PA). Step length asymmetry index (ASI) was calculated by dividing the paretic side step length by the sum of the paretic and non-paretic side step lengths, where an ASI of 0.5 indicates perfect symmetry between the paretic and non-paretic sides.^29^ The recorded ground reaction force data during the treadmill walking condition was processed (Matlab, MathWorks, MA) with a 5th order Butterworth low-pass filter with a 20 Hz cutoff frequency to smooth out the signal. Using the ground reaction force data, paretic propulsion was assessed as this variable provides a quantitative measure of the paretic side's contribution during walking.^34^ Paretic propulsion was calculated by dividing the paretic side's propulsive force by the sum of the paretic and non-paretic propulsive forces, such that a paretic propulsion of 0.5 indicates a perfect symmetry. The propulsive impulse was calculated by integrating the AGRF in the time domain. Additionally, we also assessed the maximal force generated during this propulsive phase, denoted as peak AGRF.^10^ Propulsion force and peak AGRF were normalized to the subject's body weight (%body weight). For the data obtained from each 1-min treadmill walking condition, we analyzed the gait data from the last 30 s of each trial, where all evaluated metrics were averaged over at least 10 gait cycles.

### Statistical Analysis

Means of the outcome measures for all 17 conditions (no exoskeleton, zero torque, and 15 different assistance) across five subjects were computed. To evaluate the effects of the unilateral and bilateral hip assistance on the subject's gait functions, 15 assistance conditions were categorized into five different assistance strategies:

1. Unilateral – Paretic: N0P1, N0P2, N0P3
2. Unilateral – Non-paretic: N1P0, N2P0, N3P0
3. Bilateral – Equal: N1P1, N2P2, N3P3
4. Bilateral – Paretic: N1P2, N2P3, N1P3
5. Bilateral – Non-paretic: N2P1, N3P2, N3P1

For the *Unilateral-Paretic* and *Unilateral-Non-paretic*, assistance was applied on either the paretic or non-paretic side only and for the *Bilateral-Equal*, *Bilateral-Paretic*, and *Bilateral-Non-paretic*, assistance was applied with either equal amount or differently on both sides. We performed a one-way repeated measures analysis of variance (ANOVA) on these 6 different assistance strategies (*Baseline*, *Unilateral-Paretic, Unilateral-Non-paretic, Bilateral-Equal, Bilateral-Paretic*, and *Bilateral-Non-paretic*) on the subject's walking speed, step length asymmetry, and paretic propulsion by setting an α value to 0.05 (SPSS21, IBM Corporation, Armonk, NY). Additionally, a *post-hoc* analysis with a Bonferroni correction was used to compute the statistical difference between different assistance strategies.

## Results

### Walking Speed

**Figure 3:**
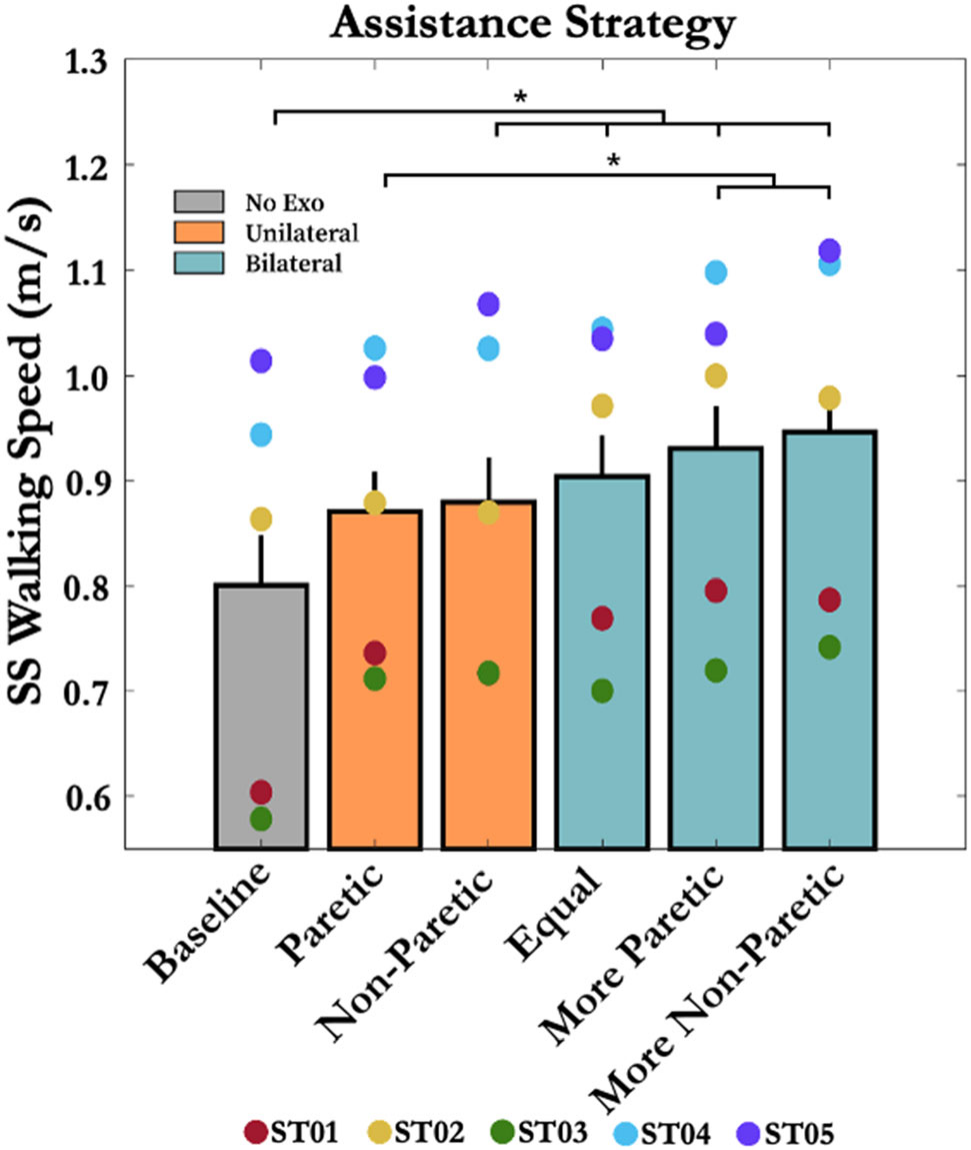
Effect of hip exoskeleton assistance strategies on stroke survivors’ self-selected walking speed. Asterisks indicate a statistical difference between conditions (*p* < 0.05). Error bars represent + 1 standard error of the mean (SEM). Unilateral and bilateral assistance strategies improved self-selected walking speed. The non-paretic bilateral strategy had the greatest increase in the user’s self-selected walking speed.

The average baseline self-selected walking speed across all subjects was 0.81 ± 0.05 m/s (mean ± SEM). Three subjects were classified as community ambulators and two subjects were classified as limited community ambulators according to functional walking category.^31^ When walking with the exoskeleton under different assistance strategies, the exoskeleton assistance significantly influenced participants' self-selected walking speed (*p* < 0.001, *η*^2^*p* = 0.54). The *Unilateral-Non-paretic, Bilateral-Equal, Bilateral-Paretic*, and *Bilateral-Non-paretic* increased the subject's self-selected walking speed on average by 11.49 ± 2.23%, 14.72 ± 2.26%, 18.16 ± 2.53%, and 19.87 ± 2.07% compared to the baseline, respectively (*p* < 0.05) (Fig. 3). Additionally, the *Bilateral-Paretic* and *Bilateral-Non-paretic* increased the subject's self-selected walking speed on average by 6.81 ± 1.10% and 8.45 ± 0.75% compared to the *Unilateral-Paretic*, respectively (*p* < 0.05).

**Figure 4:**
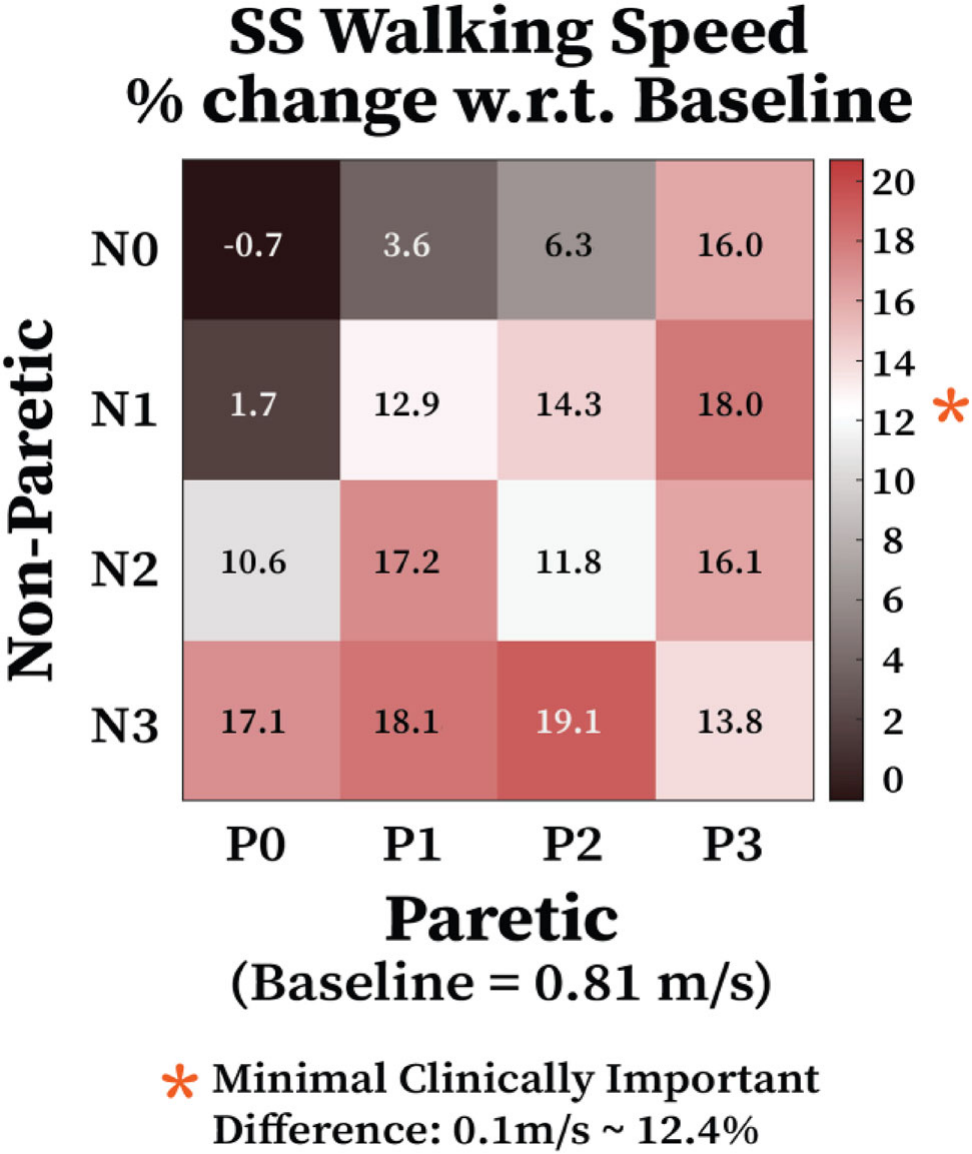
Change in the stroke survivors’ self-selected walking speed for different magnitudes of bilateral hip assistance. Each grid number indicates the percentage improvement in the participants’ walking speed compared to not wearing the exoskeleton. Asterisks indicate a minimal clinically important difference (> = 0.1 m/s threshold) in self-selected walking speed.

As shown with the average change in self-selected walking speed with respect to the baseline across conditions (Fig. 4), 10 of the 16 assistance conditions showed changes that exceeded the minimal clinically important difference (MCID). MCID for self-selected walking speed in chronic stroke survivors ranges from 0.04 m/s to 0.16 m/s.^30^ We adopted the MCID of 0.1 m/s for self-selected walking speed of substantial change as suggested,^30^ corresponding to about 12.4% change with respect to the baseline. For the assistance conditions that yield the greatest increases in self-selected walking speed in each participant, the average self-selected walking speed increase is 25% compared to the baseline, which is greater than the average self-selected walking speed increase of 19% observed in the N3P2 condition (Fig. 4).

### Step Length Asymmetry

**Figure 5:**
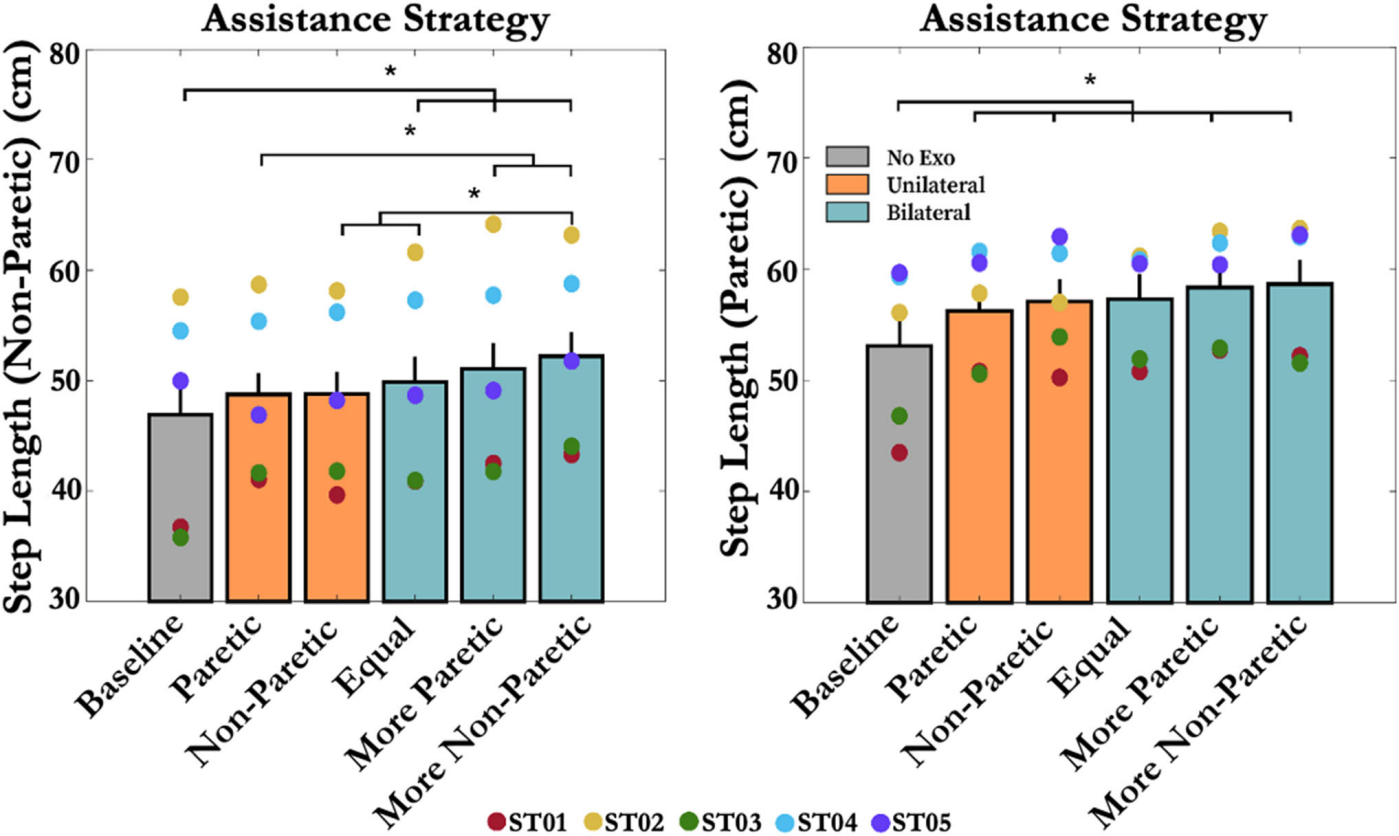
Effect of hip exoskeleton assistance strategies on stroke survivors’ step length for (left) the non-paretic and (right) paretic side. Asterisks indicate statistical difference between conditions (*p* < 0.05). Error bars represent + 1 SEM. Hip exoskeletons increased users’ step length on both limbs.

The average baseline step length ASI across all subjects was 0.53 ± 0.01, where one subject (ST02) demonstrated a shorter paretic step length (i.e., ASI < 0.5). No significant difference was observed in the step length ASI between the baseline and other five assistance strategies (*p* = 0.14, *η*^2^*p* = 0.11). The average baseline paretic step length across all subjects was 53.08 ± 2.42 cm. The *Unilateral-Paretic, Unilateral-Non-paretic, Bilateral-Equal, Bilateral-Paretic,* and *Bilateral-Non-paretic* increased the subject's paretic step length on average by 6.66 ± 1.42%, 8.27 ± 1.53%, 8.12 ± 1.46%, 10.72 ± 1.80%, and 11.07 ± 1.37% compared to the baseline, respectively (*p* < 0.05) (Fig. 5). All assistance strategies showed a greater paretic step length increase than the minimal detectable change (MDC) when compared to the baseline.

The average baseline non-paretic step length across all subjects was 46.91 ± 2.42 cm. The *Bilateral-Equal, Bilateral-Paretic*, and *Bilateral-Non-paretic* increased the subject's non-paretic step length on average by 7.06 ± 1.51%, 9.60 ± 1.76%, and 12.44 ± 1.83% compared to the baseline, respectively (*p* < 0.05). These bilateral strategies showed a greater non-paretic step length increase than the MDC when compared to the baseline. The MDC used in this study were 2.62 cm and 2.11 cm for the paretic and non-paretic step length, respectively.^16^

### Paretic Propulsion

**Figure 6:**
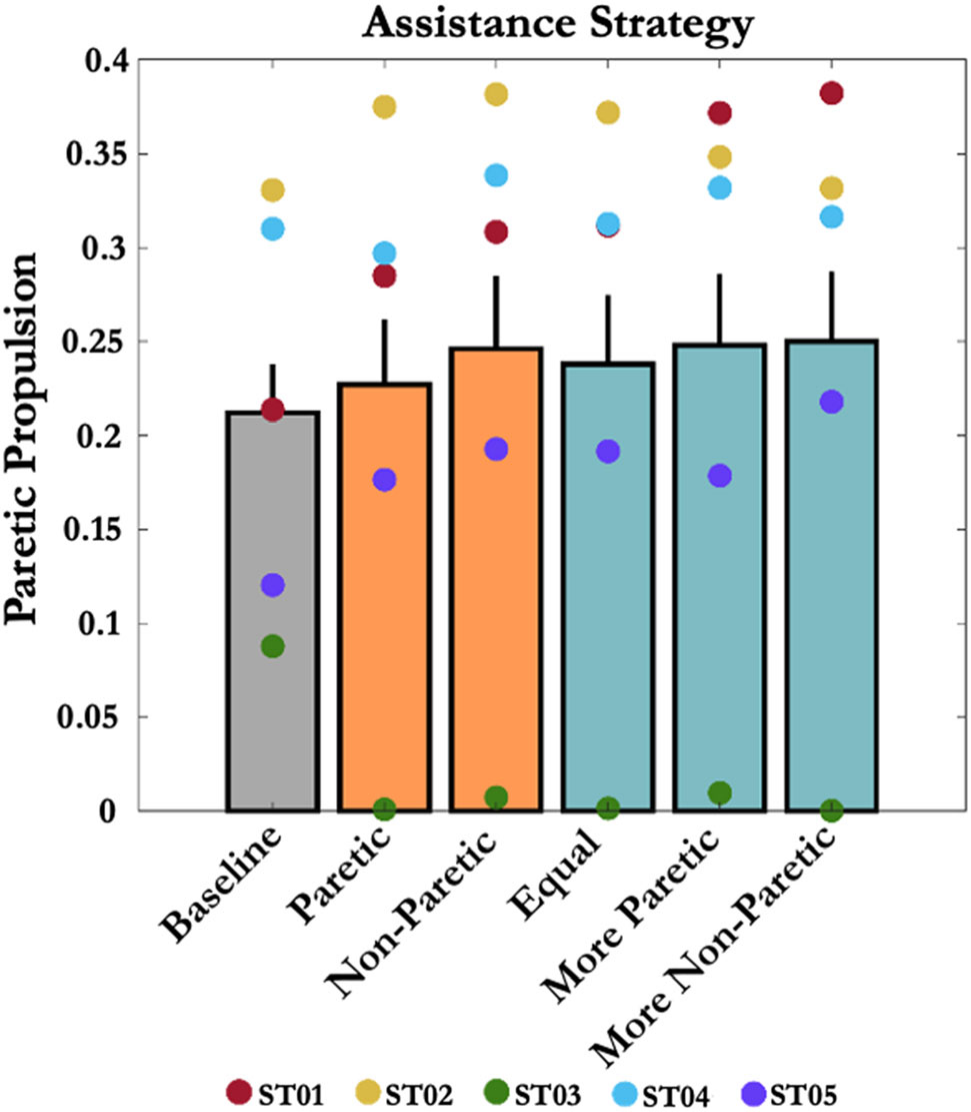
Effect of hip exoskeleton assistance strategies on stroke survivors’ paretic propulsion. A paretic propulsion of 0.5 indicates perfect symmetry. Error bars represent + 1 SEM. Hip exoskeletons had little effect on users’ propulsive symmetry.

The average baseline paretic propulsion across all subjects was 0.21 ± 0.03 (Fig. 6). The average baseline paretic peak AGRF across all subjects was 4.65 ± 0.51%BW. The average baseline non-paretic peak AGRF across all subjects was 12.6 ± 1.45%BW. No significant difference was observed in the paretic propulsion (*p* = 0.34, *η*^2^*p* = 0.39), paretic peak AGRF (*p* = 0.09, *η*^2^*p* = 0.2), and non-paretic peak AGRF (*p* = 0.13, *η*^2^*p* = 0.11) between the baseline and other five assistance strategies.

**Figure 7:**
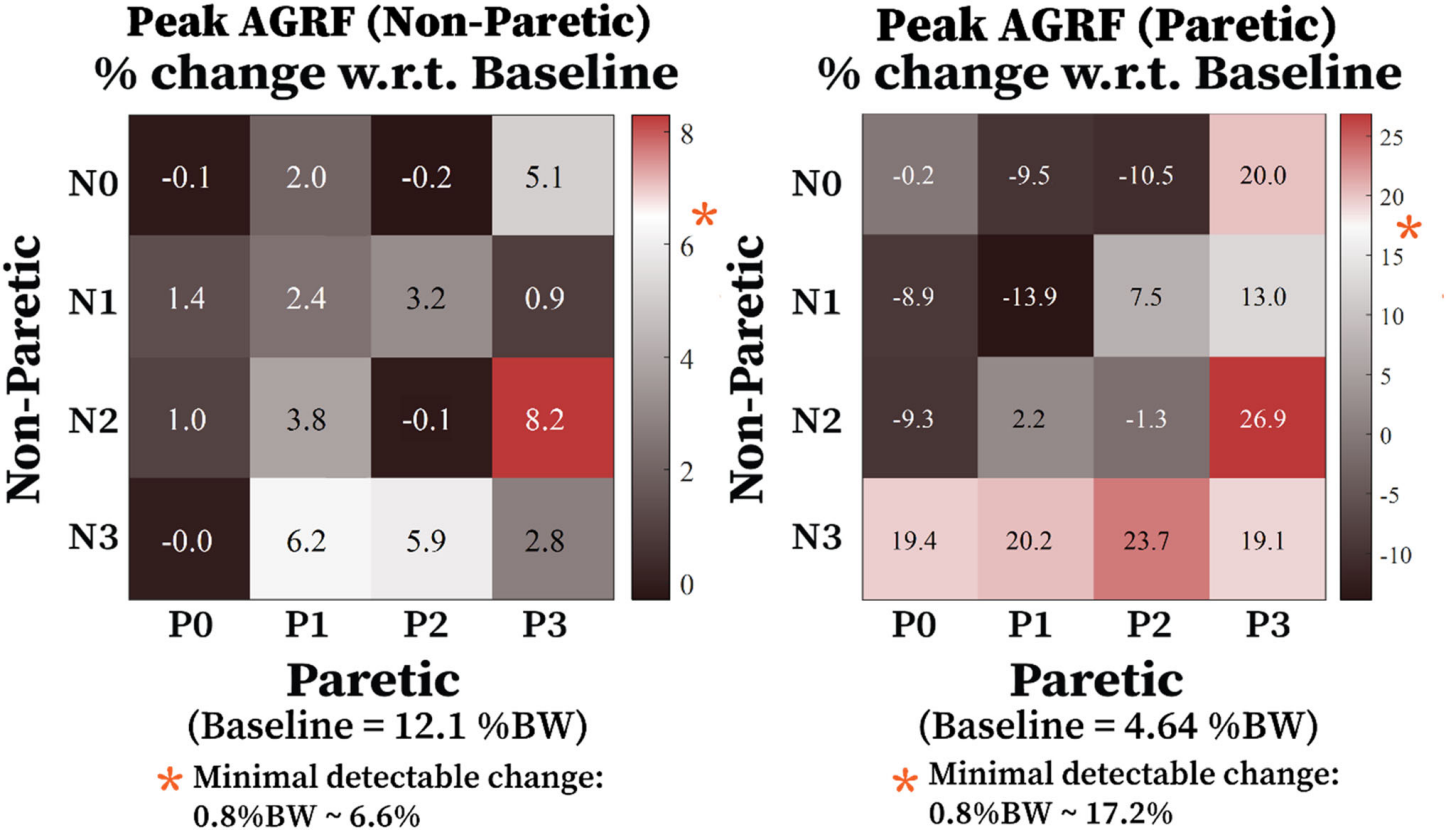
Change in the participants’ non-paretic (left) and paretic (right) AGRF for different magnitudes of bilateral hip assistance. Each grid number indicates a percentage improvement in the user’s AGRF compared to not wearing the exoskeleton. Asterisks indicate minimal detectable change (> = 0.8%BW threshold) in peak AGRF.

Considering all 16 assistance conditions, the N2P3 exhibited a greater increase in the non-paretic peak AGRF than the MDC threshold. For the paretic side, the N0P3, N2P3, N3P0, N3P1, N3P2, and N3P3 showed a greater increase in the paretic peak AGRF than the MDC threshold (Fig. 7). The MDC used in the study was 0.8%BW for both sides.^16^

## Discussion

In this study, we examined whether stroke survivors using powered hip exoskeletons could increase self-selected walking speed. We were particularly interested to know if bilateral assistance (paretic + non-paretic side) strategies would be more effective than unilateral (paretic or non-paretic side only) approaches. In line with our central hypothesis, we found that bilateral hip assistance significantly improved overground self-selected walking speed for all five stroke survivors compared to not wearing the exoskeleton (or using the exoskeleton unpowered) (Fig. 3). We also observed that the assistance strategy that provided the largest increase in self-selected walking speed was highly variable across users. Indeed, in terms of the balance between paretic and non-paretic side assistance, there was no uniformly ‘best’ strategy that increased the user’s self-selected walking speed. This indicates that there could be a benefit to individualizing gait assistance per each unique user (i.e., personalization).

On average, bilateral hip assistance increased gait speed more (17.6% increase relative to the baseline) than unilateral assistance (11.1% increase relative to the baseline) in individuals post-stroke. Specifically, asymmetrical bilateral assistance (*Bilateral-Paretic* and *Bilateral-Non-paretic*) increased self-selected walking speed more than the unilateral paretic assistance (Figs. 3 and 4). One possible reason for this is that it may be more challenging for some users to learn and adapt to exoskeleton assistance when it is assisting unilaterally. Because hemiparetic gait is asymmetric to begin with, stroke survivors may be able to adapt more quickly and effectively utilize bilateral assistance that mirrors their baseline asymmetry. Indeed, this idea was consistent with evidence from other studies indicating that unilateral hip assistance may be suboptimal^19^ given the importance of inter-limb coordination during human walking.^33, 37^

Increases in self-selected walking speed due to hip exoskeleton assistance were not accompanied by improvements in gait symmetry. Despite observed changes in step lengths on both limbs, we found no significant differences in step length ASI under any of the assistance conditions. Instead, the most effective bilateral hip assistance strategies proportionally increased *both* paretic and non-paretic step lengths (Fig. 5) and, on average, showed no significant changes in paretic propulsion (Fig. 6) across five participants. Generally, to improve asymmetry, an individual post-stroke must either increase the paretic step length or reduce the non-paretic step length. For example, even when providing more assistance on the paretic side (*Unilateral-Paretic* and *Bilateral-Paretic*), increases in stroke survivors’ step lengths remained similar on both sides. Perhaps this reflects the complicated nature of shifts in motor coordination post-stroke (e.g., merging of motor modules) that make it difficult to locally control gait parameters without global compensations.^6, 39^

Interestingly, hip exoskeleton assistance strategies that increased self-selected walking speed the most on average were bilateral and asymmetric, biased toward the non-paretic limb (Fig. 4; N3-P1&P2). In these cases, the non-paretic step length increased by 5.8% more than the unilateral assistance targeting the paretic limb only (*Unilateral-Paretic*) (Fig. 5). Taken together, these results highlight the importance of including non-paretic limb assistance in increasing self-selected walking speed for individuals post-stroke. Furthermore, the apparent lack of association between changes in gait symmetry and changes in self-selected walking speed may also suggest that symmetry per se, is not the best gait parameter to target when designing robotic assistance for stroke survivors.^23, 27, 28^ Focusing instead on propulsion on the ground may be a better target for robotic assistance intended to increase walking speed and/or reduce metabolic cost of transportation.^2^ Our results qualitatively, if not quantitatively support this idea because, of the biomechanical variables we measured, peak AGRFs tended to best reflect improvements in self-selected walking speed (i.e., Figs. 7 vs. 4).

The assistance strategy that maximized benefits in self-selected walking speed and gait biomechanics varied widely among hip exoskeleton users post-stroke. As noted earlier, across five participants we studied, asymmetrical bilateral assistance with more non-paretic assistance (e.g., N3P2 condition) was the most effective in improving hemiparetic gait measured by increased self-selected walking speed. However, this was not necessarily the most optimal strategy for each individual. For example, some stroke survivors responded best to bilateral assistance focused on the paretic side (e.g., ST02) while others did best with unilateral assistance (e.g., ST03 and ST05). Taken together, substantial inter-subject variability in the response of stroke survivors to hip exoskeleton assistance strongly suggests that individualized assistance should be considered and used to maximize benefits. This idea is consistent with other studies that highlighted the need for individualized assistance targeting the ankle joint^1, 24^ and future studies should explore this approach at the hip as well.

It is possible that lower functioning individuals post-stroke (i.e., lowest self-selected walking speed) can benefit most from hip exoskeletons. Indeed, reduced gait speed in stroke survivors is strongly associated with impaired muscle strength of the paretic hip flexors.^11, 26^ In line with this idea, we found that unilateral assistance strategies focused on compensating for weakness of the paretic limb were most effective at increasing self-selected walking speed in the two stroke participants with the lowest baseline self-selected walking speed. On the other hand, three stroke survivors with fastest self-selected walking speed at the baseline showed no clinically meaningful difference in self-selected walking speed with unilateral assistance on the paretic limb (Fig. 1). Further study is needed to relate the effectiveness of hip exoskeleton assistance to the severity and neuromechanical characteristics of gait impairment at baseline. For example, it may be possible to grossly tune assistance strategies based on the motor strategies exhibited by each individual user.^41^

Our study has some limitations worth noting. First, we did not examine a large number of participants and this limited the power of our statistical analyses. Nevertheless, we found a clear general trend that the bilateral assistance has a greater benefit than the unilateral assistance and intra-participant variability in the details of the assistance strategy (e.g., balance between paretic and non-paretic side) is paramount. Next, due to the constraints in total experimental duration, we focused on examining the effects of different hip assistance levels on both sides (one baseline + 15 conditions with different assistance magnitudes on both sides). The assistance timing was not rigorously tuned to each participant and was fixed across all assistance conditions. Future studies should consider the effect of hip exoskeleton assistance timing to maximize improvements in gait function on individuals post-stroke. Furthermore, while the main biomechanical analysis in this study was focused on the AGRF, we collected joint kinematics (Supp. Figure 1 in Supplemental Document) during the experiment. A future study can expand our analysis and investigate the effect of assistance level on inter-joint coordination.

## Conclusions

In this study, we investigated the effect of different hip exoskeleton assistance strategies on stroke survivors’ self-selected walking speed and gait biomechanics. Our results indicate that the bilateral hip assistance is an effective strategy that can significantly increase the user’s paretic step length by 10% and self-selected walking speed by 17.6%, compared to the baseline. In general, we found that increasing assistance bilaterally improved the overall effect of the exoskeleton. However, the magnitude balance between the paretic and non-paretic side was varied, highlighting the need for personalized bilateral assistance strategies to maximize benefit per individual.

## Supplementary Information

Below is the link to the electronic supplementary material.

Supplementary file1 (PDF 1077 kb).
