# Supplementary material for: Effects of Bilateral Assistance for Hemiparetic Gait Post-Stroke Using a Powered Hip Exoskeleton
Source: Ann Biomed Eng. 2022 Aug 13;51(2):410–21. doi: 10.1007/s10439-022-03041-9 (PMC9867666; doi:10.1007/s10439-022-03041-9)

Supplemental Document: Individual joint kinematics across assistance conditions

Experimental setup: In addition to the original data recordings, standard motion capture data was collected to calculate the joint kinematics (hip, knee, and ankle) across different exoskeleton assistance conditions. Different colors represent different assistance strategies, and a black dashed line represents the baseline condition of walking with the exoskeleton powered off (zero torque). The Left and right graph represent the subject's paretic and non-paretic side, respectively.

Supp. Fig. 1. Hip, knee, and ankle joint position across 5 different exoskeleton assistance strategies for paretic and non-paretic side

Hip joint position for ST01

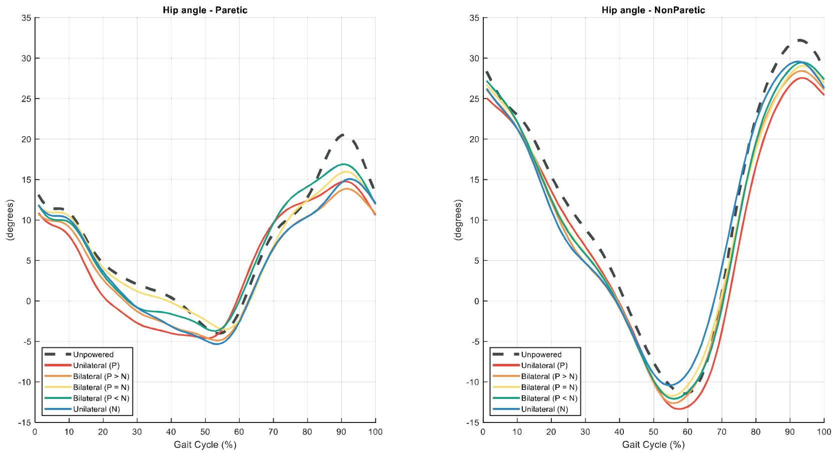

Knee joint position for ST01

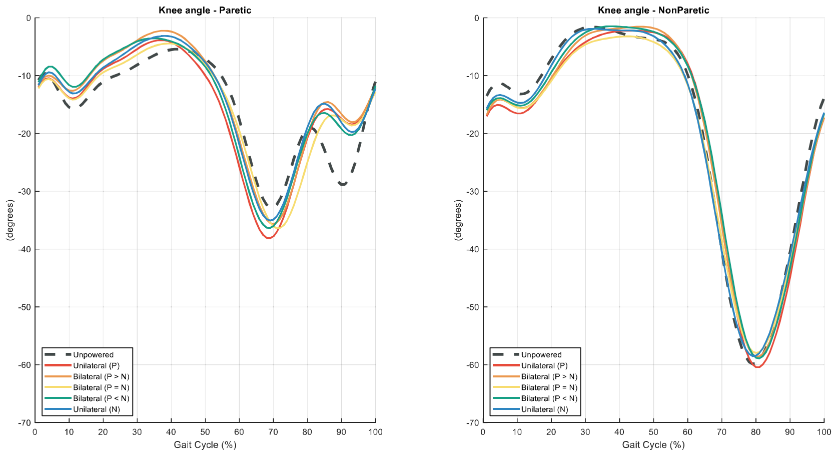

Ankle joint position for ST01

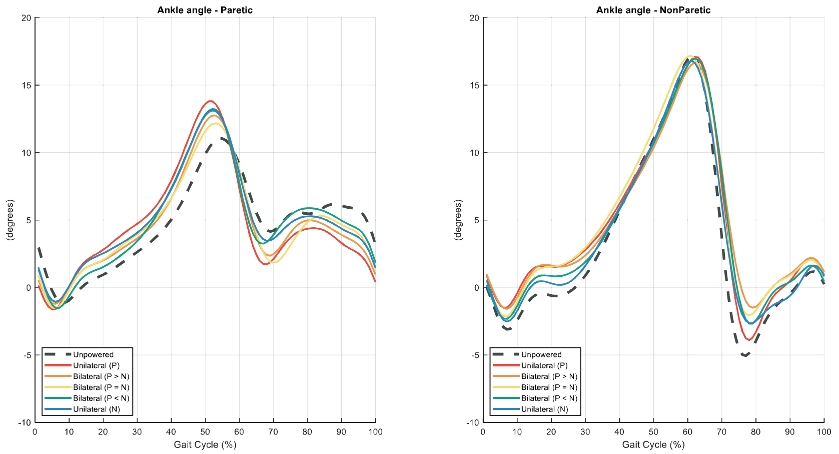

Hip joint position for ST02

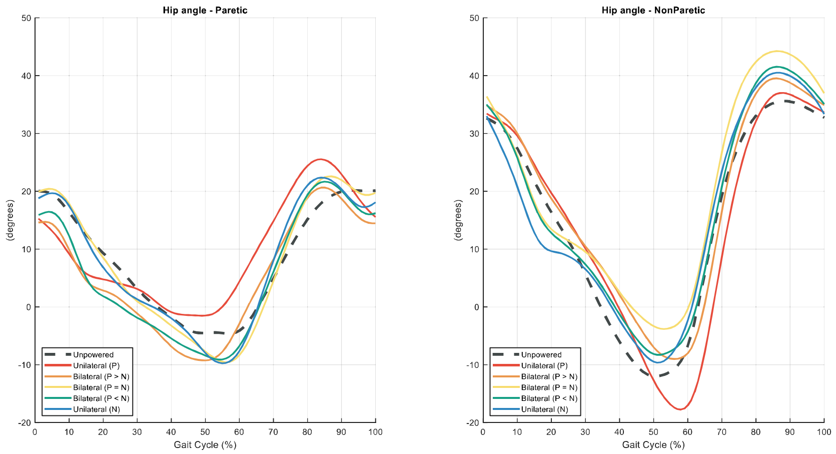

Knee joint position for ST02

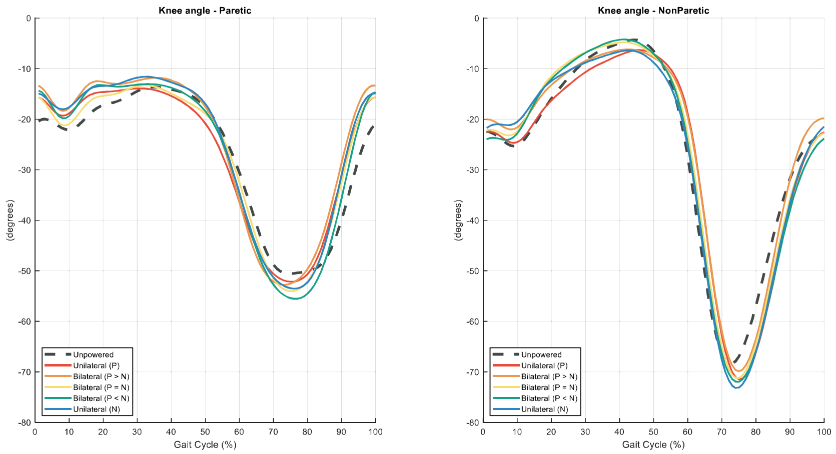

Ankle joint position for ST02

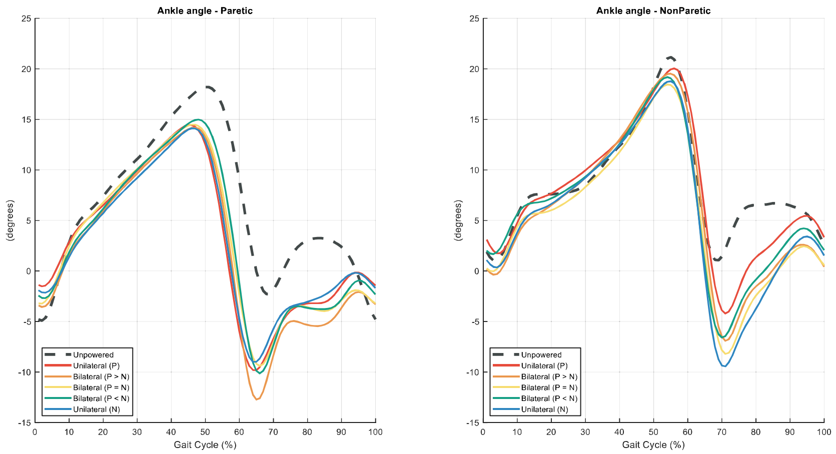

Hip joint position for ST03

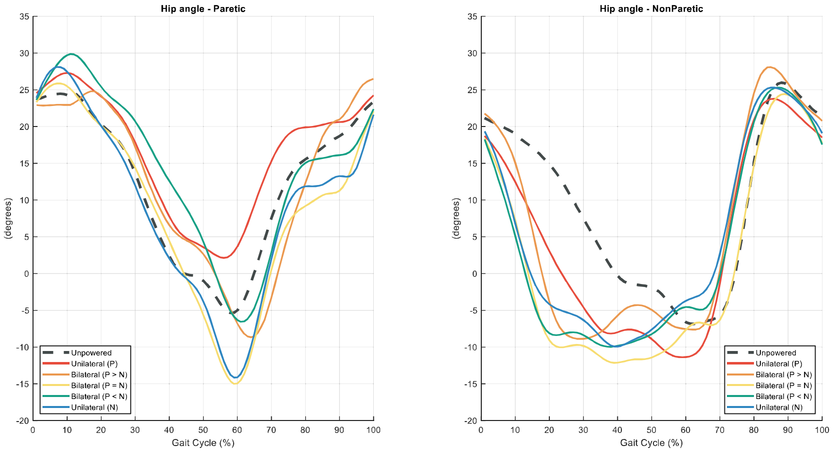

Knee joint position for ST03

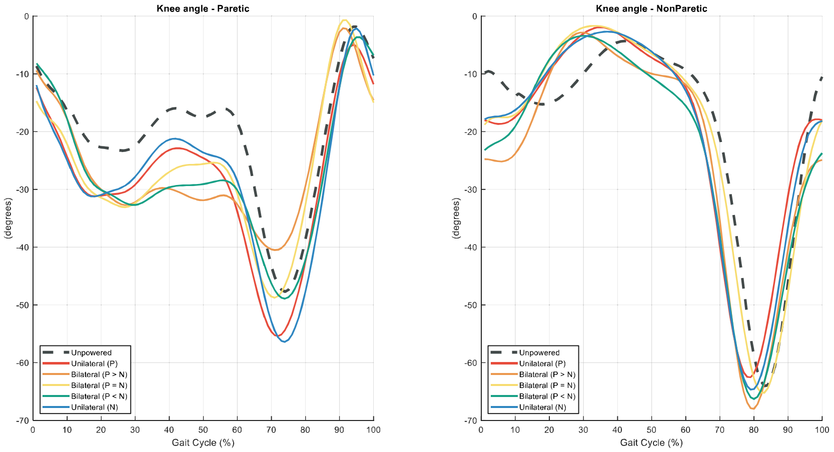

Ankle joint position for ST03

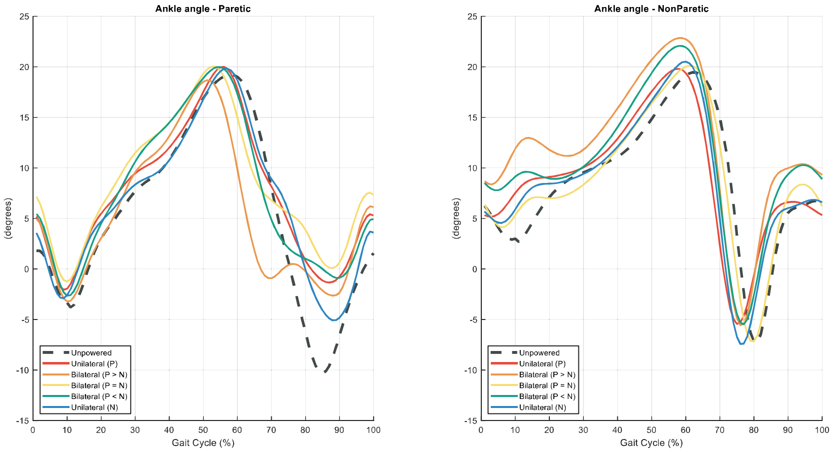

Hip joint position for ST04

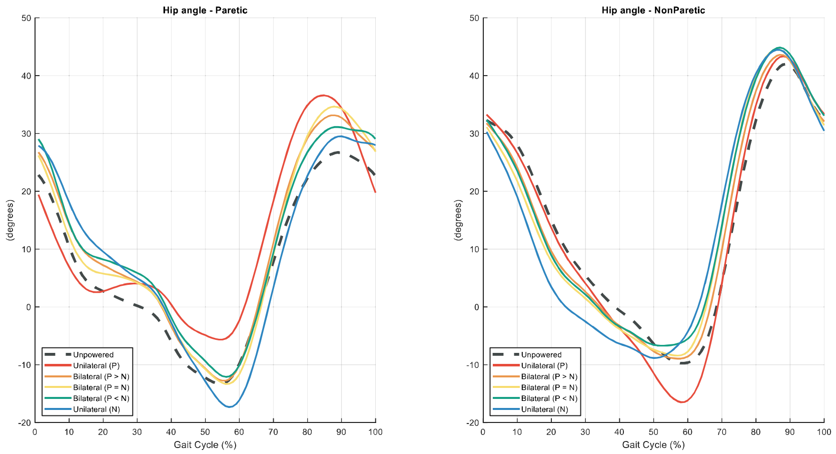

Knee joint position for ST04

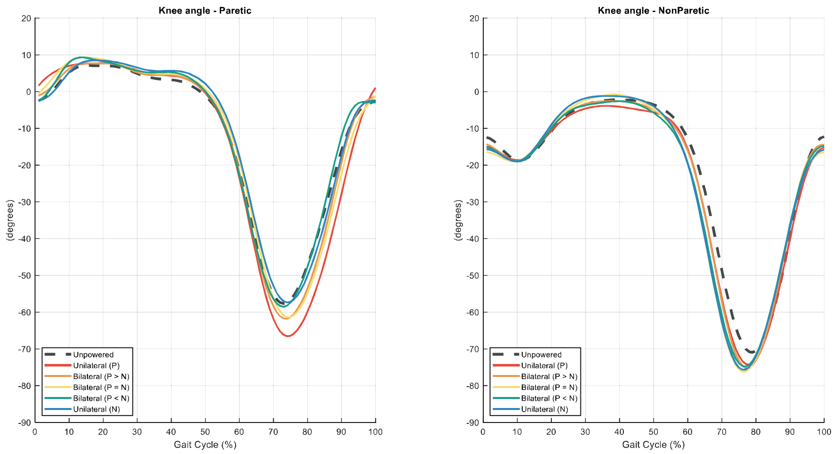

Ankle joint position for ST04

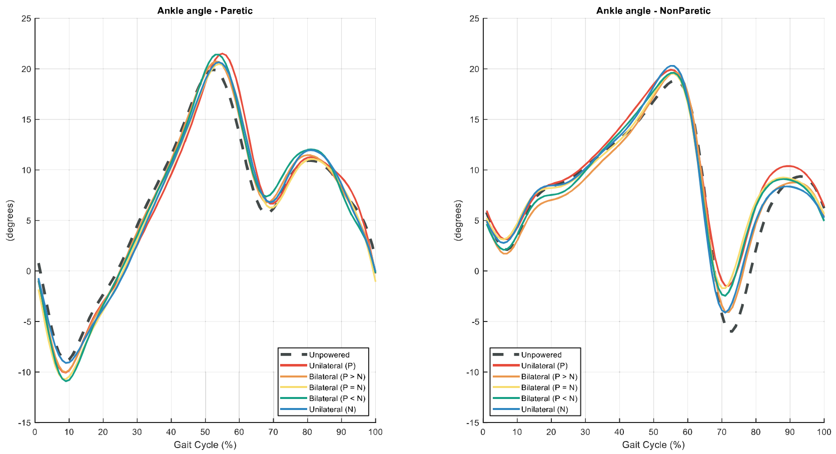

Hip joint position for ST05

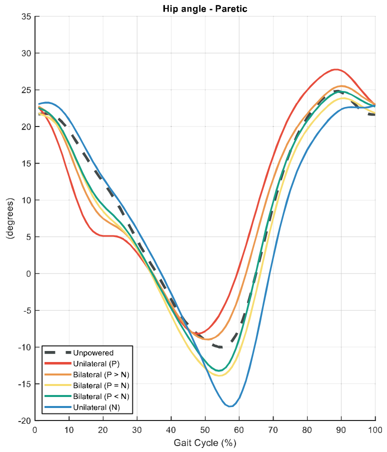

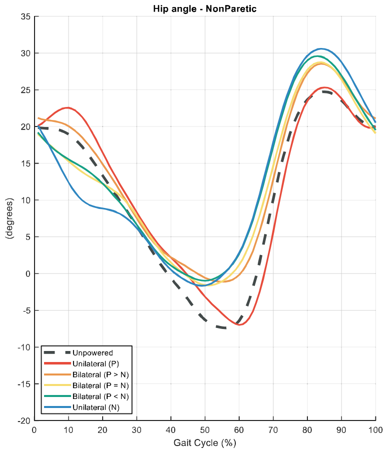

Knee joint position for ST05

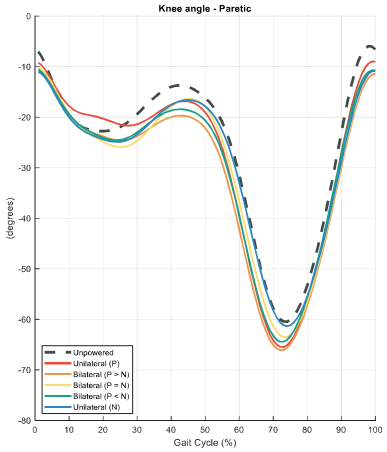

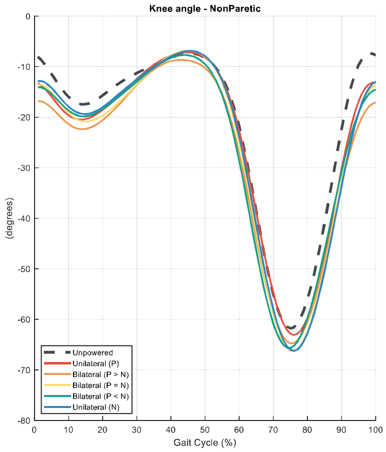

Ankle joint position for ST05

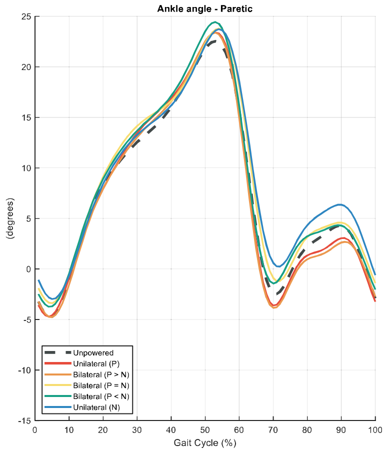

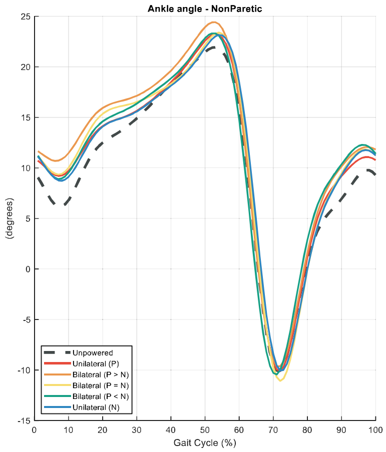

Supplement: Supplementary file 1 — Supplementary file1 (PDF 1077 kb). [file 10439_2022_3041_MOESM1_ESM.pdf]
